# Comparing various protocols of human and bovine ovarian tissue decellularization to prepare extracellular matrix-alginate scaffold for better follicle development in vitro

**DOI:** 10.1186/s12896-020-00658-3

**Published:** 2021-01-20

**Authors:** Hossein Nikniaz, Zahra Zandieh, Mohammad Nouri, Neda Daei-farshbaf, Reza Aflatoonian, Mazaher Gholipourmalekabadi, Seyed Behnamedin Jameie

**Affiliations:** 1 Department of Anatomical sciences, School of Medicine, Iran University of Medical Sciences, Tehran, Iran; 2 Department of Reproductive Biology, Faculty of Advanced Medical Sciences, Tabriz University of Medical Sciences, Tabriz, Iran; 3 Department of Endocrinology and Female Infertility at Reproductive Biomedicine Research Center, Royan Institute for Reproductive Biomedicine, ACECR, Tehran, Iran; 4 Cellular and Molecular Research Center, Iran University of Medical Sciences, Tehran, Iran; 5 Department of Tissue Engineering & Regenerative Medicine, Faculty of Advanced Technologies in Medicine, Iran University of Medical Sciences, Tehran, Iran; 6 Neuroscience Research Center (NRC), Iran University of Medical Sciences, Tehran, Iran

**Keywords:** Decellularization, Scaffold, Ovary, Fertility preservation

## Abstract

**Background:**

Nowadays, the number of cancer survivors is significantly increasing as a result of efficient chemo/radio therapeutic treatments. Female cancer survivors may suffer from decreased fertility. In this regard, different fertility preservation techniques were developed. Artificial ovary is one of these methods suggested by several scientific groups. Decellularized ovarian cortex has been introduced as a scaffold in the field of human fertility preservation. This study was carried out to compare decellularization of the ovarian scaffold by various protocols and evaluate the follicle survival in extracellular matrix (ECM)-alginate scaffold.

**Results:**

The micrographs of H&E and DAPI staining confirmed successful decellularization of the ovarian cortex in all experimental groups, but residual DNA content in SDS-Triton group was significantly higher than other groups (*P* < 0.05). SEM images demonstrated that complex fiber network and porosity structure were maintained in all groups. Furthermore, elastin and collagen fibers were observed in all groups after decellularization process. MTT test revealed higher cytobiocompatibility of the SDS-Triton-Ammonium and SDS-Triton decellularized scaffolds compared with SDS groups. Compared to the transferred follicles into the sodium alginate (81%), 85.9% of the transferred follicles into the decellularized scaffold were viable after 7 days of cultivation (*P* = 0.04).

**Conclusion:**

Although all the decellularization procedures was effective in removal of cells from ovarian cortex, SDS-Triton-Ammonium group showed less residual DNA content with higher cytobiocompatibility for follicles when compared with other groups. In addition, the scaffold made from ovarian tissues decellularized using SDS-Triton-Ammonium and sodium alginate is suggested as a potential 3D substrate for in vitro culture of follicles for fertility preservation.

## Background

Nowadays, the number of cancer survivors is significantly increasing as a result of efficient chemo/radio therapeutic treatments [1]. But female cancer survivors may suffer from decreased fertility. Thus, fertility preservation is one of the most important issues in these patients. In this regard, different fertility preservation techniques, such as ovarian shielding or transposition, embryo and oocyte cryopreservation, ovarian tissue cryopreservation, and artificial ovary were developed [2, 3]. However, due to the risk of transmission of cancer cells by transplanting cryopreserved ovarian tissue, artificial ovary and in vitro maturation were suggested by several scientific groups [4–6]. In recent techniques, ovarian follicles were isolated from the surrounding tissue and transferred into the scaffold to maintain mechanical support [7]. Studies demonstrated that follicle growth and the survival rate depend on type, structure, and rigidity of the scaffold and its ability to enhance vascularization after transplantation [8, 9]. So, scientific groups attempt to find ideal scaffold to support follicle development [10]. To reach this aim, different kinds of natural and synthetic matrixes were examined. Polyethylene glycol (PEG) was the only synthetic scaffold ever used but varieties of natural scaffolds were examined. Alginate, collagen, and fibrin hydrogels transmit nutrients to the follicle rapidly. In recent years, extracellular matrix (ECM) was used as a scaffold in the field of reproductive medicine [9, 11–13]. ECM scaffold is obtained by decellularizing the tissue. Decellularization is performed by physical, chemical, or combinative methods [12]. The type of decellularization agents and duration of exposure have effect on structure and biocompatibility of the ECM scaffold [11, 14]. Therefore, different techniques of decellularization were tested to obtain optimized ovarian ECM scaffolds [9, 12, 13]. Sodium dodecyl sulfate (SDS) human and bovine ovarian tissue has been decellularized by SDS, SDS-Triton, and sodium lauryl ester sulfate (SLE). Although SDS is the ionic surfactant and a prevalent agent used in the decellularization process and successfully eliminates cells’ nuclear materials, it can be harmful to the structural of ECM and signaling proteins [15]. Furthermore, it is a cytotoxic agent and irremovable due to its ionic nature [11]. Therefore, researchers are attempting to reduce the exposure time and concentration of the SDS and increase the biocompatibility of the scaffold through combining it with other decellularization materials. Triton X-100 is a nonionic surfactant commonly used as a decellularizing material [16]. Liu et al. decreased SDS exposure time and decellularized ovarian tissue by SDS-Triton protocol [12]. But elimination of residual DNA was lower than SDS alone. Ammonium hydroxide is a decellularizing agent commonly used in combination with SDS [11]. Ammonium retains glycosaminoglycans (GAGs) and viscoelasticity of the decellularized tissue by counteracting the negative charge of collagen [17].

In addition to decellularization materials, the type of tissue used as a scaffold is important in the growth and differentiation of cells. Recently, ovarian cortical decellularized tissue has been used as a scaffold in regenerative medicine. Laronda et al. demonstrated that by culturing ovarian cells on to the decellularized scaffold, ovarian cells can produce estrogen after transplantation and can initiate puberty in ovariectomized mice [9]. Hassanpoure et al. cultured primary ovarian cells on the decellularized ovarian scaffolds and reconstructed follicle-like structures after transplantation [13]. However, the viability of follicles into the decellularized scaffold was not assessed. So, for the first time, in the present study, the isolated ovarian follicles were 3D cultured into the SDS-Triton-Ammonium decellularized ovarian scaffold using alginate hydrogel and the viability of follicles was tested.

So, in the present study, various decellularization protocols were compared to find optimized ovarian tissue decellularization method in terms of biocompatibility and residiual DNA. Furthermore**,** for the first time, isolated preantral follicles were cultured into the decellularized ovarian scaffold and the viability of follicles was tested.

## Results

### Decellularization of bovine ovarian cortex

**Fig. 1:**
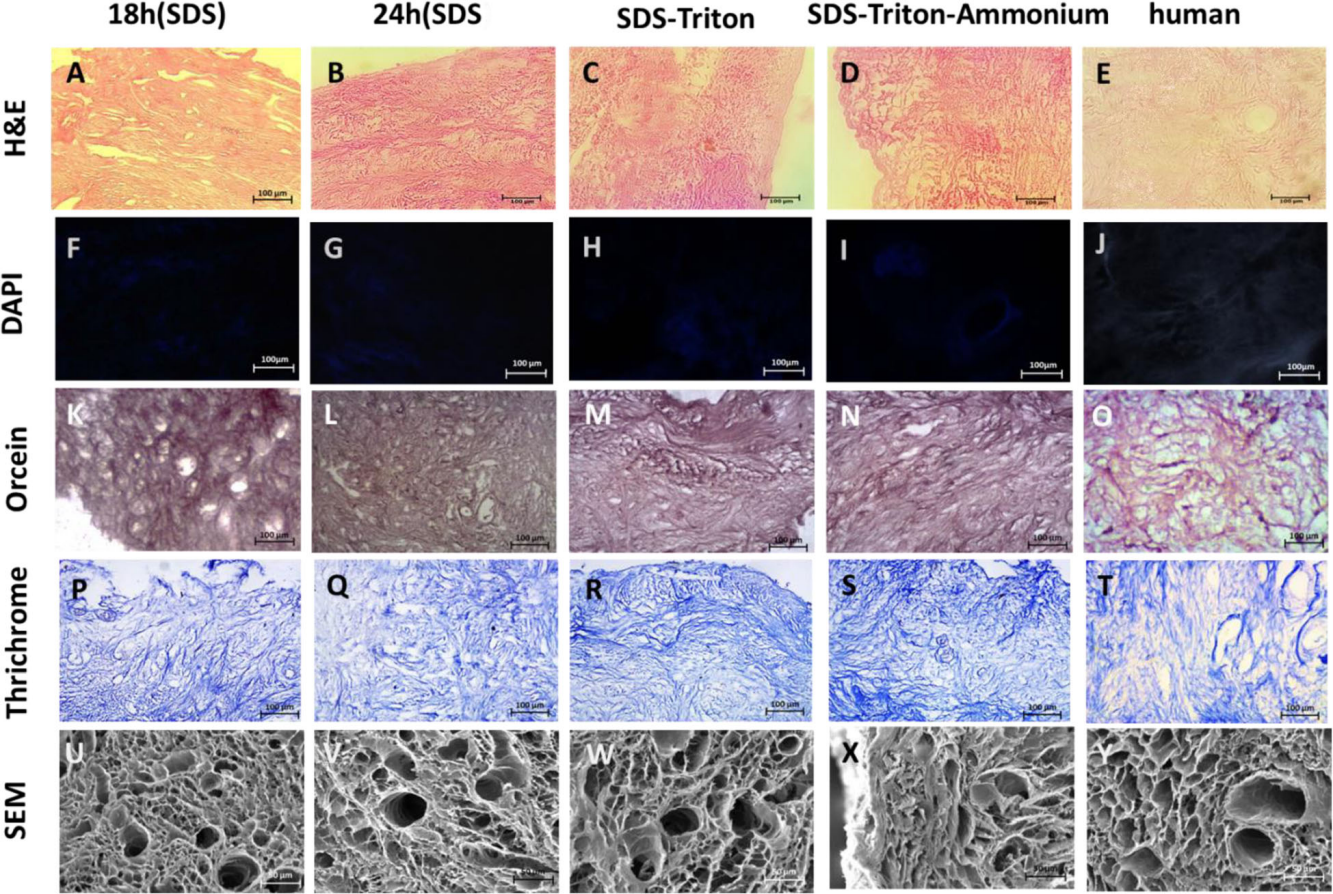
Extracellular matrix structure and composition preservation. Decellularization of the ovarian cortex confirmed by DAPI staining (**a**-**d**). Preservation of scaffold microarchitecture presented by SEM (**U-Y**). Orcein staining (**K-O**) and Masson’s Trichrome staining (**P-T**) demonstrate the presence of elastic and collagen fibers, respectively. Scale bars: 100 μm

The H&E staining results showed that ovarian tissues were completely decellularized in all groups (Fig. 1a-e). Furthermore, no residual nuclei were observed by DAPI staining (Fig. 1f-j). The results of data quantification with DNA content kit showed that the residual DNA in SDS-Triton group was significantly higher than SDS-18 h, SDS-24 h, and SDS-T-A groups (*P* < 0.05) (Table 1).

**Table 1:** Comparison of decellularization and cytotoxicity of decellularization methods

Decellularization methods		SDS 18 Mean ± SD	SDS24 Mean ± SD	SDS-T Mean ± SD	SDS-T-A Mean ± SD
**Residual DNA (%)**		10.4 ± 2.35 ^a^	9.13 ± 0.85 ^b^	15.2 ± 1.9 ^c^	7.16 ± 1.6 ^d^
**MTT test assay (%)**	**24 Hours**	87.33% ± 28.57	68% ± 20.51	80% ± 9.84	96.33% ± 12.74
	**7 days**	43.33 ± 10.50 ^e^	14% ± 8.32 ^f^	77.66% ± 14.64 ^g^	75% ± 15.71 ^h^

Significant difference between a and c (*P* < 0.028), b and c (*P* < 0.054), c and d (*P* < 0.011), e and f (*P* < 0.03), e and g (*P* < 0.014), e and h (*P* < 0.021), f and g (*P* < 0.001), and f and h (*P* < 0.001). The experiments where performed in three replication and results were expressed as mean ± standard deviation

Staining of elastic fibers and collagen proteins with orcein (Fig. 1 K-O) and trichrome (Fig. 1 P-T, respectively, revealed that the ECM components remained intact after decellularization process. A complex fiber network with porous and uniform microstructure was clearly seen in all the decellularized samples under SEM (Fig. 1 U-Y). Along with light microscopy results, no visible cells were found in the decellularized groups. Furthermore, after the cells removal, the vessels, follicles, empty spaces and pores were preserved. In higher magnification, intact collagen networks were observed with no damage during the decellularization process.

### Decellularized human ovarian cortex analysis

The human ovarian cortex was decellularized with SDS-Triton-Ammonium. The results of H&E and DAPI staining demonstrated that the human ovarian cortex was decellularized completely but DNA content analysis showed that it contained 24.1% ± 3.7% of the DNA matched with intact samples. Fiber structure and porosity were preserved as the bovine ovarian cortex. However, in the human ovarian cortex, the pore size was smaller and the collagen fibers were denser than the bovine ovary (Fig. 1Y).

### MTT assay

To compare the cytotoxicity effect of decellularization methods, the viability of fibroblast cells was assessed with and without scaffolds after one and 7 days. The results showed that the viability of cells was not significantly different between groups after 1 day, but the percentage of cell viability in SDS groups (18 and 24 h) was significantly lower than SDS-T and SDS-T-A groups after 7 days. Moreover, there was no significant difference between SDS-T and SDS-T-A groups (Table 1). Cells viability of human decellularized scaffold was 81.1%. There was no significant difference between cell viability of human and bovine decellularized tissue treated with SDS-Triton-Ammonium.

### Viability of follicles after 7 days of cultivation in decellularized ovarian tissue using hydrogel

**Fig. 2:**
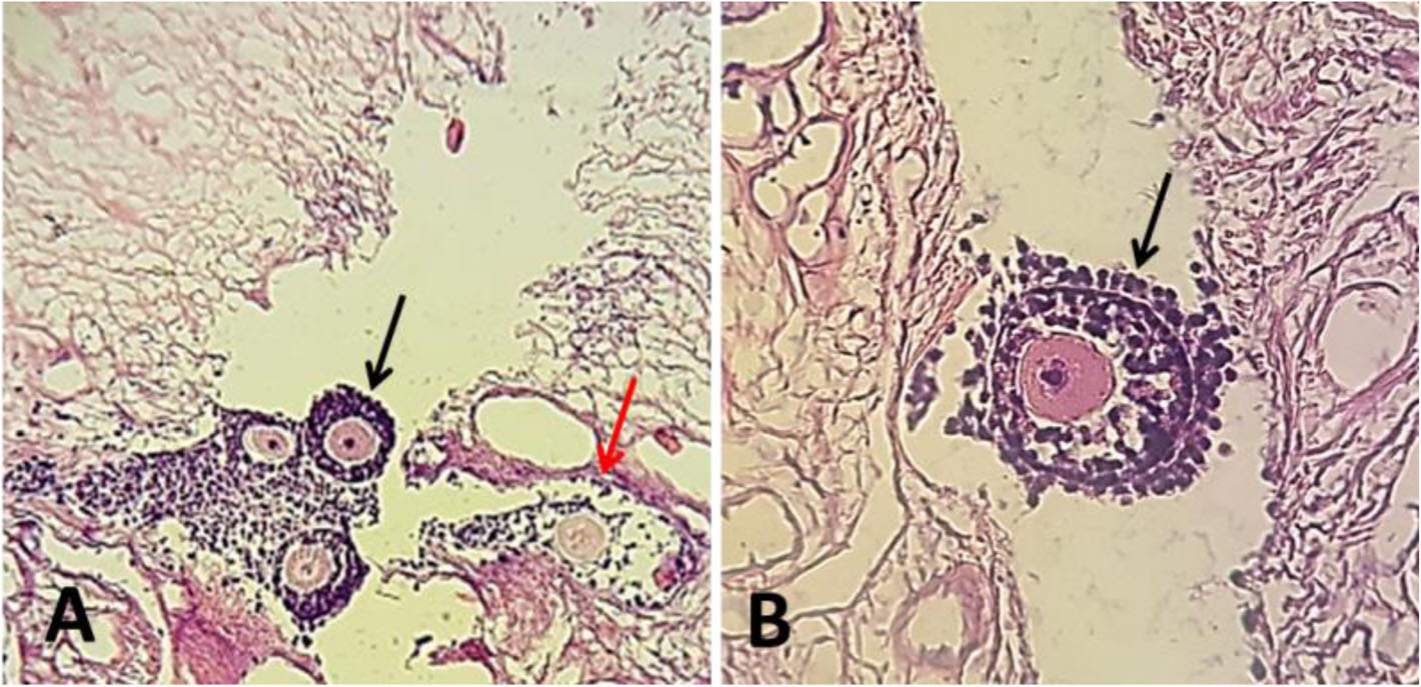
Mice follicles encapsulated in the decellularized matrix after 7 days of cultivation. Histologically, normal (black arro) and degenerated follicles (red arrow) were found after histological analysis

To assess viability of follicles after isolation, follicles were stained by Trypan blue. Out of 50 isolated preantral follicles, 89% had a high quality and 11% had a low quality. Also, no dead follicles were found. After transferring the follicles into the decellularized scaffolds, their histology was assessed by H&E staining (Fig. 2). Based on the results, no significant difference was observed in the recovery rate of preantral follicle cultured into the SDS-Triton-Ammonium decellularized scaffold [85.9% (220/256)] and alginate alone [81% (166/205)] after 7 days (*P* = 0.04).

## Discussion

Currently, the application of decellularized tissue in regenerative medicine has been considered because of its biological activity, three-dimensional structure, and capacity for providing the suitable conditions for cell adhesion, migration, differentiation, and proliferation [18]. In this regard, in the present study we used ECM scaffold matrix and showed that although there was no significant difference in the structure of decellularized scaffold, the elimination of residual DNA was different and cell viability could be affected by different decellularization methods. Besides, this study demonstrated that similar to alginate alone, the composition of the decellularized scaffold with alginate could preserve follicles viability in vitro.

The ECM is an organized network of proteins and GAGs. It is a dynamic structure which can remodel throughout follicle development, ovulation, and atresia [19]. ECM compartment affects estrogen and progesterone production and plays a role in the viability of GCs [19]. Studies demonstrated that GCs cultured on ECM proliferate more rapidly than cells cultured on plastic culture dish due to connection with ECM ligands and the presence of cellular growth factors stored in the ECM [18]. Furthermore, the rates of oocyte maturation in alginate with ECM compartments were higher than alginate alone [20]. Decellularized tissue can improve follicles viability and growth by providing natural ECM composition, growth factors, and porous structure [21]. Due to the features of the decellularized tissue, it can be used as a scaffold for follicle culture.

Decellularization methods can affect the structure of the ECM, maintenance of growth factors, amount of residual DNA, and the cellular bioactivity in the scaffold [12]. The results of the present study showed that decellularization with SDS-T and SDS-T-A had the highest and lowest residual DNA, respectively. Triton eliminates residual DNA better than SDS, especially when combined with ammonium [11, 22]. Also, Triton led to less damage to ECM structure and better cell viability when compared with SDS [11]. In line with previous results, in the present study, cell viability was higher on scaffolds with a shorter exposure to SDS [15].

The GCs environment can affect gene expression. GCs cultured on ECM protein-coated culture dish produce a higher level of estrogen and have better proliferation and differentiation rates but follicle 3D culture system is crucial to inhibit GCs detachment from the oocyte and improve follicle survival and development [23, 24]. Alginate is one of the most commonly used scaffolds for follicles 3D culture. Alginate is non-toxic, biocompatible hydrogel, and easy to prepare [25]. In order to use ECM proteins in the follicle three-dimensional culture system, ECM molecules were combined with alginate to create synthetic ECM hydrogel. The results of follicle culture in alginate-ECM protein hydrogel showed that it had a positive effect on estrogen production, follicle development, and oocyte maturation [21, 26].

In Intact decellular tissue matrix, porosity and protein composition was not changed so in vivo and in vitro biological activity was remained. Therefore, in the present study, we attempted not only to preserve the overall structure of the ovarian cortex, but also maintain 3D conditions for the follicles. To this aim, we made a hole inside the scaffold with an insulin needle and inserted the follicles with the alginate into the hole. This type of scaffold can be suitable for in vitro culture because porous structure is permeable for culture media. Furthermore, intact ECM could imitate follicle native tissue microenvironment by providing growth factors. On the other hand, alginate is a well-known non-toxic hydrogel for three-dimensional follicle culture. It’s permeable for large molecules ranging from 5 nm to 200 nm; so, hormones such as follicle-stimulating hormone (FSH) can diffuse easily into the hydrogel within 15 min [1]. The results of this study showed that the viability of the follicles in decellularized ovarian scaffold was not decreased when compared with control group. In the study group, follicles were surrounded by a thin layer of alginate. As follicles cultured in the 3D environment can easily access the culture media and benefit from decellularized tissue, the viability of follicles was not decreased compared with alginate alone. Due to the effect of decellularized scaffold on gene expression in follicles, it can be better choice for follicle culture in vitro [23, 24].

The results of the present study should be interpreted considering the following limitation. The study was performed in vitro just for 7 days. Thus, long-term culture and extra analysis such as maturation of oocyte and grafting in ovariectomized mice are needed.

In conclusion, decellularization of ovarian cortex by SDS-Triton-Ammonium protocol not only has less residual DNA compared with other methods, but also is biocompatible for follicle culture; in addition, it is effective in human ovarian cortex decellularization.

Murine isolated preantral follicles survive and develop when composed with the alginate-ECM scaffold. Due to the characteristics of the decellularized scaffold, seeding, vascularization, and consequently follicle viability in vivo would be better than hydrogel scaffolds and they could be used as a suitable scaffold in the artificial ovary. Application of other natural hydrogels such as ECM hydrogels instead of alginate is suggested for further studies.

## Materials and methods

### Animal husbandry and care

The present study was approved by the Ethics Committee on Human and Animal Research, Iran University of Medical Sciences, Tehran, Iran (ir.iums.fmd.rec.1398.031). In this study, the Naval Medical Research Institute (NMRI) female mice were maintained in a temperature-controlled room with a 12-h light/12-h dark cycle.

### Bovine ovarian tissue acquisition and decellularization

Bovine ovaries were collected from a local slaughterhouse and transported to the lab. Upon arrival, the connective tissue and fat were isolated and ovaries were washed with phosphate-buffered saline (PBS) and then were frozen at − 80 °C for further treatments.

**Fig. 3:**
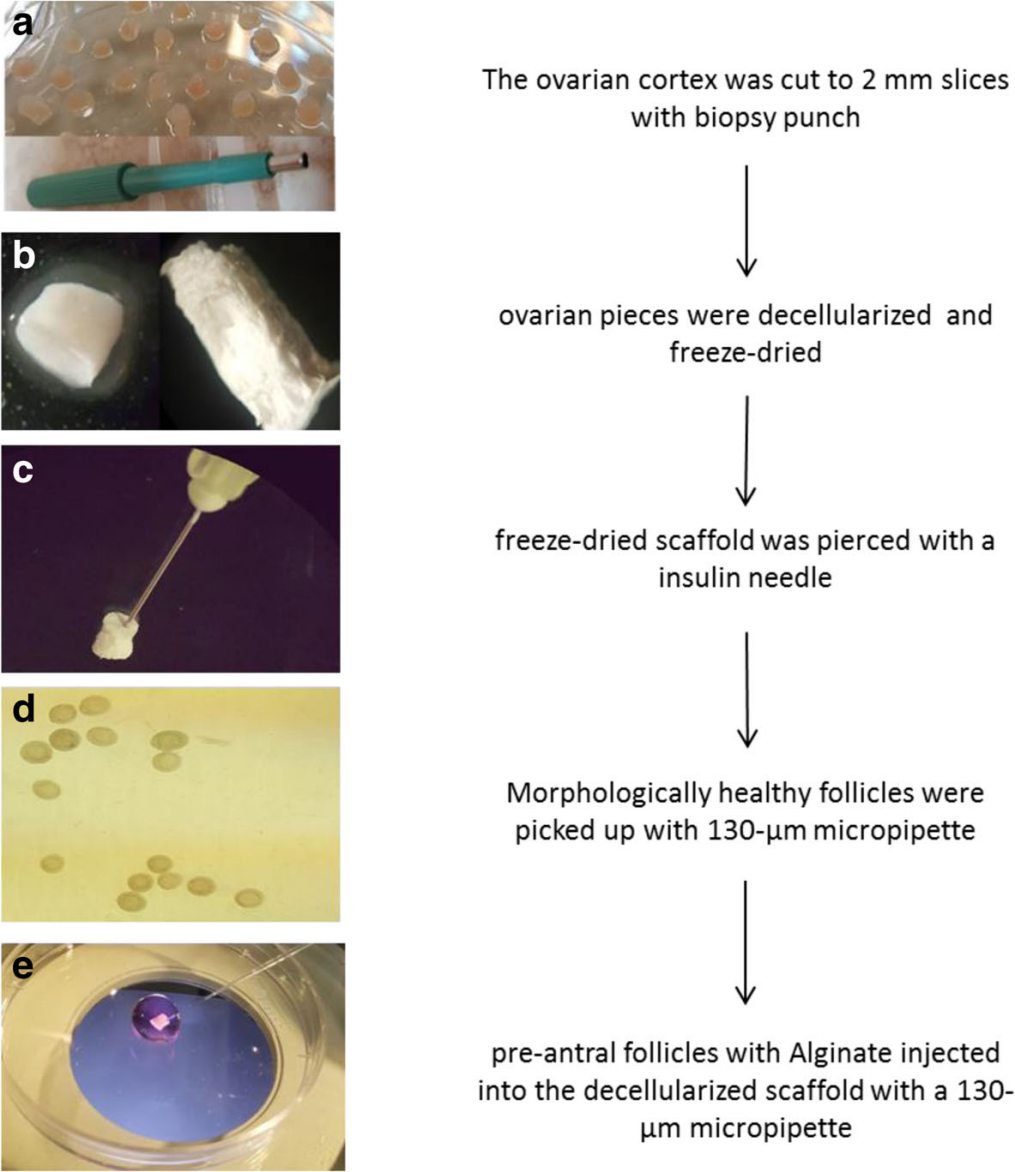
Ovarian cortex was cut to 2 mm pieces and then decellularized with SDS-T-A and freeze-dried. Scaffolds were pierced with insulin needle and alginate with preamtral follicles were injected into the scaffold

After thawing, each ovary was butterflied along the hilus, and then medulla was removed with a sharp blade. The remaining cortex was cut into equal parts with a two-millimeter biopsy punch (Fig. 3a). For decellularization process, the pieces were frozen and thawed three times at − 80 °C and 37 °C, respectively. Then, ovarian pieces were decellularized with four different methods.

In the first method, ovarian pieces were treated with 0.1% SDS for 24 h (SDS-24 h group); in the second method, the pieces were treated with 0.1% SDS for 18 h (SDS-18 h group); in the third method, the pieces were decellularized with 0.5% SDS for 3 h and 1% Triton for 9 h (SDS-T group); and in the last method, the pieces were decellularized with 0.5% SDS for 2 h and then treated with composition of 1% Triton and 0.1% Ammonium hydroxide for 22 h (SDS-T-A group). After the decellularization process, all samples were washed in deionized water for 24 h and dried with freezing and lyophilizing process (Fig. 3b).

### Human ovarian tissue acquisition and transport

Four Human ovarian tissue was obtained from females (age range: 34–45 years) undergoing ovarian tissue removal. A written informed consent was obtained from all participants.

The ovaries were transported to the laboratory at 4 °C within 1 h. Human ovarian pieces were prepared in the same way as the bovine ovary. They were decellularized with the same method of SDS-T-A group, which had the best results in the bovine ovary.

### Tissue fixation and histological analysis

The ovarian cortex and decellularized pieces were fixed in 4% paraformaldehyde overnight. All fixed tissues and scaffolds were processed by an automated tissue processor (Leica) and embedded in paraffin. Five-micrometer sections were prepared and stained with hematoxylin and eosin (H&E). Decellularized scaffolds were stained by DAPI (Sigma, USA) to confirm the decellularization process.

### DNA content analysis

DNA count assay was performed to investigate residual nuclear material of decellularized scaffold by QIAamp® DNA Blood and Tissue Mini Kit (QiagenGmbH, Hilden, Germany) according to the instructions. The extracted DNA materials (ng/μL) were analyzed by spectrophotometer (the optical density (OD) at γ = 260 nm) using the NanoDrop® ND-1000 (Nanodrop Technologies Inc., Wilmington, DE, USA).

### Extracellular matrix (ECM) analysis

The presence of elastin and collagen fibers was evaluated by Orcein staining and Masson’s trichrome staining, respectively.

### Scanning electron microscope (SEM)

To examine the structure of decellularized scaffolds, all lyophilized ovarian pieces were prepared for SEM. Firstly, ovarian pieces were put into the liquid nitrogen and cut with a sharp blade for exposing the inner region. Then, they were mounted with gold-palladium using a Humen VII sputter coater (Anatech Ltd., Alexandria, VA, US).

### MTT assay

To determine cytocompatibility of decellularized ovarian samples in vitro, 5 × 10^6^ mice fibroblast cells were cultured in DMEM/F12 medium (Shell Max; A2930) containing 10% fetal bovine serum (FBS, Gibco, Paisley UK), 100 U/mL penicillin, and 100 μg/mL streptomycin (Gibco) in 96-well dish for 24 h at 5% CO_2_ and 37 °C. After 24 h, decellularized scaffolds of each group were put into the wells. For the 2D control group, fibroblast cells were cultured without scaffold. After 1 and 7 days of cultivation, 20 μl of media was removed and 20 μl of the MTT solution (1 mg/mL of MTT in PBS) was added and the cultures were incubated for 4 h. After 4 h, culturing media was completely removed and Formazan crystals were dissolved by 300 μL dimethyl sulfoxide (Sigma-Aldrich) for 15 min. The optical density (OD) was measured by spectrophotometer (DANA-DA3200, Iran) at a wavelength of 560 nm to define the metabolic activity level of the cells.

### Isolation of preantral follicles from mouse ovaries

Eleven 14-day-old female NMRI mice were provided from the Center of Experimental and Comparative Study, Iran University of Medical Sciences, Tehran, Iran. Mice were killed by cervical dislocation. Ovaries were removed aseptically and placed in 40 μl isolation media (Dulbecco’s phosphate-buffered saline (DPBS)) supplemented with 10% fetal bovine serum (FBS; Sigma-Aldrich), 100 mIU/ml penicillin, and 100 mg/ml streptomycin (Sigma Aldrich) in 4°^C^ and separated from the connective tissue. Then, ovaries were placed in 30 μl isolation media and prenatal follicles were dissected mechanically with a 29-gauge needle. Two hundred fifty six morphologically healthy follicles were picked up with 130-μm micropipette (Flexipet; Cook) [27]. Follicles were picked up within 45 min under a stereomicroscope (Leica; Van Hopplynus Instruments). The follicular population was characterized after isolation.

### Follicle viability test

After follicle isolation, the viability of isolated preantral follicles was assessed by trypan blue dye. To this aim, 15 μl of 0.4% trypan blue was added to 300 μl of isolated media containing 50 preantral follicles and incubated for 10 min at room temperature. Afterward, follicles were classified as high viable, low viable, or dead based on oocyte and granulosa cell (GC) viability. High viable follicles had less than 10% dead GCs, low viable follicles had 10–50% dead GCs, and dead follicles had a dead oocyte.

### Alginate hydrogel preparation

Sodium alginate (Sigma; MO; USA) was diluted in deionized water to a final concentration of 5% (w/v). Organic impurities were removed with activated charcoal (0.5 g charcoal/ g alginate). Following charcoal treatment, the alginate solution was filtered through 0.22 μM filters under sterile conditions after 24 h. Finally, 5% alginate was diluted to 1% (w/v) alginate with sterile 1% PBS.

### Transferring follicle into the decellularized ovarian scaffold and alginate

In the study group, SDS-Triton-Ammonium decellularized ovarian cortex was used as a scaffold to 3D culture in vitro. Ten scaffolds were pierced with a 27-gage insulin needle under sterile conditions and put into the culture media (Fig. 3c). Isolated preantral follicles (24–30/ scaffold) were transferred into the 20 μl 1% alginate (final concentration = 0.7%) (Fig. 3d). Alginate with preantral follicles injected into the decellularized scaffold with a 130-μm micropipette (Flexipet; Cook) and then scaffolds suspended into the encapsulation solution (140 mM NaCl / 50 mM CaCl2) for 3 min (Fig. 3e).

In the control group, a droplet of culture media containing isolated preantral follicle was transferred to droplets (40 μl) of alginate solution (final concentration = 0.7%). To form the beads, the droplets were slowly released into the crosslinking bath consisting of calcium solution at 37 °C. Then, the beads were removed and washed in PBS and transferred to the culture media.

### In vitro culture

Scaffolds with follicles were cultured in a 24-well plate containing 500 μl αMEM supplemented with 10 mIU ml^− 1^ recombinant follicle stimulating hormone (Merck; Darmstadt, Germany) 3 mg ml^− 1^ bovine serum albumin (BSA; Sigma-Aldrich), 5 mg ml^− 1^ insulin, 5 mg ml^− 1^ transferrin, and 5 mg ml^− 1^ selenium (Gibco, UK) for 7 days.

### Post-culture analysis

After fixation and paraffin embed of the scaffold, five-micrometer serial sections were prepared and H&E staining was performed. Follicles were counted in all slides.

### Statistical analysis

Statistical analysis was performed with SPSS version 22 software (SPSS, Chicago, IL) and included means and SDs. Differences among group means for the various variables were evaluated by one-way analysis of variance (one-way ANOVA) followed by post-hoc test (Tukey’s HSD test) for multiple comparisons. A difference was considered significant if the *p*-value was < 0.05.

## Data Availability

The data are available with reasonable request from the corresponding author.

## Abbreviations

ECM: Extracellular matrix
SEM: Scanning electron microscope
NMRI: Naval Medical Research Institute
PEG: Polyethylene glycol
SDS: Sodium dodecyl sulfate
SLE: Sodium lauryl ester sulfate
GAGs: Glycosaminoglycans
FSH: Follicle-stimulating hormone
PBS: Phosphate-buffered saline
OD: Optical density
DPBS: Dulbecco’s phosphate-buffered saline
GC: Granulosa cell
BSA: Bovine serum albumin
